# Increase in Th17 and T-reg Lymphocytes and Decrease of IL22 Correlate with the Recovery Phase of Acute EAE IN Rat

**DOI:** 10.1371/journal.pone.0027473

**Published:** 2011-11-07

**Authors:** Beatriz Almolda, Manuela Costa, Maria Montoya, Berta González, Bernardo Castellano

**Affiliations:** 1 Unit of Histology, Department of Cell Biology, Physiology and Immunology, Institute of Neuroscience. Universitat Autònoma de Barcelona, Bellaterra, Spain; 2 Servei de Cultius Cel.lulars, Producció d'Anticossos i Citometria. Universitat Autònoma de Barcelona, Bellaterra, Spain; 3 Centre de Recerca en Sanitat Animal, UAB-IRTA, Campus de la Universitat Autònoma de Barcelona, Bellaterra, Barcelona, Spain; Institute Biomedical Research August Pi Sunyer (IDIBAPS) - Hospital Clinic of Barcelona, Spain

## Abstract

Experimental autoimmune encephalomyelitis (EAE), a well-established model of multiple sclerosis, is characterised by microglial activation and lymphocyte infiltration. Induction of EAE in Lewis rats produces an acute monophasic disease characterised by a single peak of disability followed by a spontaneous and complete recovery and a subsequent tolerance to further immunizations. In the current study we have performed a detailed analysis of the dynamics of different lymphocyte populations and cytokine profile along the induction, peak, recovery and post-recovery phases in this paradigm. MBP-injected rats were sacrificed attending exclusively to their clinical score, and the different populations of T-lymphocytes as well as the dynamics of different pro- and anti-inflammatory cytokines were analysed in the spinal cord by flow cytometry, immunohistochemistry and ELISA. Our results revealed that, during the induction and peak phases, in parallel to an increase in symptomatology, the number of CD3+ and CD4+ cells increased progressively, showing a Th1 phenotype, but unexpectedly during recovery, although clinical signs progressively decreased, the number and proportion of CD3+ and CD4+ populations remained unaltered. Interestingly, during this recovery phase, we observed a marked decrease of Th1 and an important increase in Th17 and T-reg cells. Moreover, our results indicate a specific cytokine expression profile along the EAE course characterized by no changes of IL10 and IL17 levels, decrease of IL21 on the peak, and high IL22 levels during the induction and peak phases that markedly decrease during recovery. In summary, these results revealed the existence of a specific pattern of lymphocyte infiltration and cytokine secretion along the different phases of the acute EAE model in Lewis rat that differs from those already described in chronic or relapsing-remitting mouse models, where Th17-cells were found mostly during the peak, suggesting a specific role of these lymphocytes and cytokines in the evolution of this acute EAE model.

## Introduction

Experimental autoimmune encephalomyelitis (EAE) is a useful animal model for the study of multiple sclerosis (MS) that is produced by the immunisation of susceptible animals with myelin proteins. Among the different EAE models, immunisation of susceptible Lewis rats with Myelin Basic Protein (MBP) induced an acute monophasic disease characterised by weight loss and hindlimb paralysis, followed by a spontaneous recovery, after which animals became resistant to further immunisations with MBP [1], [2]. This EAE model is of special interest because provides a unique tool to study the cellular and molecular mechanisms involved not only in the induction of the disease, but more interestingly, those participating in the recovery process and the establishment of immunological tolerance.

Although there are a wide number of studies published about different aspects of EAE in Lewis rats, the major part of these studies are focused on specific time-points, mainly at the peak of the disease, whereas molecular and cellular changes taking place during the induction and recovery phases remain poorly defined. To address this lack of information and gain insights into the mechanisms involved in the initiation and resolution of the pathological process, in the last years we have performed a series of detailed studies in which animals were sacrificed attending exclusively to their clinical score along the different phases characteristics of this model. In these studies we have reported changes in the morphology and phenotype of microglial cells, in terms of antigen presenting capacity, along the different phases of EAE showing that during the induction and peak phases, microglia exhibited a phenotype of immature dendritic cells, characterised by MHC-class I and class-II expression, no co-stimulatory molecules and CD1 expression, whereas during the recovery and even post-recovery phases, activated microglia maintained MHC expression and a subpopulation of cells expressed B7.2 [3] and/or CD4 [4].

In addition to microglial reactivity it is well established that T-cell infiltration is a crucial feature of EAE [5]. Specifically, CD4+ Th1 cells are widely accepted as the key players in leading to the immune response associated with EAE [6]. However, in the last years, upon the emergence of new subsets of CD4+ T-cells with different cytokine profiles and functions [7], [8], [9], this classical assumption has been reconsidered and nowadays it is suggested that, in addition to Th1 cells, other subtypes of CD4+ T-cells may also be involved in EAE pathogenesis. Indeed, it has recently been demonstrated that injection of Th17 lymphocytes, a new characterised subset of CD4+ T-cells [10], [11], are able to induce EAE in mice [12]. Moreover, accumulation of T-regulatory Foxp3+ cells (T-regs) was reported within the mouse CNS concurrent with EAE recovery [13], [14], [15]. Although an important number of reports can be found in the literature about infiltration of T-cells in different EAE models [16], [17], [18], [19], [20], [21], [22], [23], it is important to take into account that major part of these studies are focused specially in the peak of the disease, and do not characterized the different subtypes of T-cells in the induction and recovery phases. Moreover, some of these studies analysed only changes occurring in lymphatic organs, especially spleen and lymph nodes [17], [19], or described changes in the CD4+T-cell population but not in the subtypes of these cells [16], [21], [22], [23]. Nevertheless, little is known about the dynamics of the specific lymphocyte subtypes inside the spinal cord along the course of the disease.

In this way, the aim of the present study was to perform a careful analysis of CD4+ T-cell subsets dynamics and their cytokine profile along the induction, peak, recovery and post-recovery phases in the acute EAE model induced in Lewis rats. Our results indicate that the population of T-cells remained unaltered during all the phases of the acute EAE but showing specific phenotypes during the induction and recovery phases. Interestingly, and in contrast to studies in EAE mice models, in this acute model the population of Th17 cells was higher during the recovery phase.

## Materials and Methods

### Ethics Statement

All experimental animal work was conducted according to Spanish regulations in agreement with European Union directives (86/609/CEE, 91/628/CEE i 92/65/CEE) and was approved by the Ethical Committee of the Autonomous University of Barcelona (CEEA-UAB 569).

### Animals and EAE induction

A total of 140 female Lewis rats (180 g/200 g) susceptible to develop experimental autoimmune encephalomyelitis (EAE) were purchased from Charles River (France) and maintained with food and water *ad libitum* in a 12 h light/dark cycle.

EAE was induced by the injection in both hindlimbs of an emulsion containing 100 µg myelin basic protein (MBP) (Sigma, USA, Ref. M2295) in Complete Freud's Adjuvant (CFA) (Ref. 0638; Difco; USA) and 0.2 mg of *Mycobacterium tuberculosis* (*M. tuberculosis*) H37 Ra (Ref. 3114; Difco; USA). Animals injected with vehicle solution were used as control (sham).

The presence of clinical signs was evaluated daily in all animals, using the following clinical score test: 0, no clinical signs; 0.5, partial loss of tail tonus; 1, tail paralysis; 2, paraparesis of hindlimb; 3, paraplegia; 4, tetraparesis; 5, tetraplegia and 6, death. Paralysed animals were afforded easier access to food and water.

### Experimental groups

As in previous studies [3], [4], EAE-induced animals were sacrificed according to their clinical score, at different phases along the EAE course, as detailed: 1) before the appearance of symptomatology (2, 4, 6 and 8 days post-immunisation); 2) during the induction phase: at score 0.5, score 1 and score 2; 3) at the peak of the disease (score 3); 4) during the recovery phase: at score 2 of recovery (score 2R), score 1 of recovery (score 1R) and 0 of recovery (score 0R) and 5) during the post-recovery phase, at 28, 32, 40 and 90 days post-immunisation (referred to as score 0R**–**28dpi, score 0R**–**32 dpi, score 0R**–**40 dpi and score 0R**–**90 dpi). A total of 56 EAE-induced rats and 7 shams injected with vehicle were used for flow cytometry and ELISA studies. For immunohistochemistry, 68 EAE-induced rats and 9 sham animals were processed.

### Flow cytometry analysis

As already described in previous studies [4], for flow cytometry analysis, animals were anesthetised with 0.015 ml/g ketamine (80 mg/kg)/xylazyne (10 mg/kg) and intracardially perfused with phosphate buffer solution (PBS). Quickly, the entire spinal cord was dissected out and the meninges were carefully removed. In order to obtain cell suspensions, tissue was dissociated through meshes of 140 µm and 70 µm and digested with a mixture of DNase I (28 U/ml; 10 104 159 001; Roche) and collagenase (0.2 mg/ml; LS004194, Worthington). Subsequently, each cellular suspension was centrifuged for 20 min at 600 g at room temperature in a discontinuous-density Percoll gradient (17-0891-02; Amersham-Pharmacia) between 1.08 g/ml and 1.03 g/ml. Myelin in the upper layer was removed. Cells in the interphase and in the upper-phase were collected, washed in PBS +2% serum and labelled during 30 min at 4°C with different combinations of the following surface markers: anti-CD4-PECy5 (1∶400; 554839; BD Pharmingen; San Diego, CA), anti-CD4-APC.Cy7 (1∶400; 201518; Biolegend), anti-CD3-FITC (1∶400; 557354; BD Pharmingen, San Diego, CA), and anti-CD45RC-PE (1∶400; 554888; BD Pharmingen, San Diego, CA). Subsequently, for the detection of intracellular markers, samples were permeabilised for 40 min using the Foxp3 staining buffer set (00-5523-00; eBiosciences; San Diego, CA) and labelled for 30 min at 4°C with anti-Tbet-PerCP.Cy5.5 (1∶400; 45-5825; eBiosciences; San Diego, CA), anti-GATA3-PE (1∶400; 560074; BD Pharmingen; San Diego, CA), anti-RORγ-APC (1∶400; IMG-6275G; IMGENEX; San Diego, CA) and anti-Foxp3-PE.Cy7 (1∶400; 25**–**5773; eBiosciences; San Diego, CA) following the instructions specified in the manufacturer's protocol. In parallel, isotype-matched control antibodies for the different fluorochromes were used as negative controls and spleen samples as positive control. Spleen samples were also used to determine the gates for CD3+ and CD3+CD4+cells. In order to perform the quantification of the total number of cells, a known volume of fluorescence beads (CytoCountTM, S2366, DakoCytomation) was added and mixed with each sample. Finally, cells were acquired using a FACScalibur or FACsCanto flow cytometer (Becton Dickinson; San Jose, CA), and the results were analysed using FlowJo® software. Quantification of total number of cells was performed following the methodology specified in the manufacturer's data-sheet (CytoCountTM, S2366, DakoCytomation). Spinal cords from each animal in both sham and EAE groups were analyzed separately. Only in the case of the transcription factors study spinal cords were pooled (a minimum of three animals per clinical score).

### Tissue processing for histological analysis

Animals processed for immunohistochemistry were sacrificed under deep anaesthesia (ketamine/xylazyne) as previously described and perfused intracardially with 4% paraformaldehyde in 0.1 M PBS (pH 7.4)+5% sucrose. Spinal cords (cervical and thoracic part) were dissected out immediately, postfixed for 4 h at 4°C in the same fixative, and series of parallel longitudinal sections (40 µm thick) were obtained using a Leica VT 1000 S vibratome. Series were stored at −20°C in the Olmos antifreeze solution until their later use.

### Single immunohistochemistry

Some parallel free-floating vibratome sections were processed for the visualisation of different subtypes of lymphocytes: CD3 for all T-cell populations and CD4 for T-helper cells. After endogenous peroxidase blocking with 2% H_2_O_2_ in 70% methanol for 10 min, sections were blocked in 0.05 M Tris-buffered saline (TBS), pH 7.4, containing 10% foetal calf serum, 3% bovine serum albumine (BSA) and 1% Triton X-100 for 1 h. Afterwards, sections were incubated overnight at 4°C with either anti-CD3 (1∶500; A0452; Dakopatts, Denmark) or anti-CD4 (1∶1000; MCA55G; AbD Serotec) antibodies diluted in the same blocking solution. Sections incubated in media lacking the primary antibody were used as negative controls, and spleen sections as positive control. After washes with TBS +1% Triton, sections were incubated at room temperature for 1 h with either biotinylated anti-mouse secondary antibody (1∶500; BA-2001; Vector Laboratories, Inc; Burlingame, CA) or biotinylated anti-rabbit secondary antibody (1∶500; BA-1000; Vector Laboratories, Inc; Burlingame, CA). After 1 h in streptavidin-peroxidase (1∶500; SA-5004; Vector Laboratories, Inc; Burlingame, CA), the reaction was visualised by incubating the sections in a DAB kit (SK-4100; Vector Laboratories, Inc; Burlingame, CA) following the manufacturer's instructions. Finally, sections were mounted on slides, some of them counterstained with toluidine blue, dehydrated in alchohol and after xylene treatment, coverslipped in DPX. Sections were analysed and photographed with DXM 1200F Nikon digital camera joined to a Nikon Eclipse 80i microscope.

### Double immunohistochemistry

Double-immunolabelling was carried out by firstly processing the sections with either CD3 or CD4 immunolabelling as described above, but using, as secondary antibodies, AlexaFluor® 488-conjugated anti-rabbit (1∶1000, A-21206; Molecular Probes) in the case of CD3, or AlexaFluor® 555-conjugated anti-mouse (1∶1000, A31570; Molecular Probes) in the case of CD4. After several washes, these sections were incubated overnight at 4°C with either rabbit anti-Iba1 (1∶3000; 019**–**19741; Wako), mouse anti-CD4 (1∶1000; MCA55G; AbD Serotec), rabbit anti-Tbet for demonstration of Th1 cells (1∶1000; sc-21003; Santa Cruz Biotechnology), rabbit-anti-Foxp3 for demonstration of T-reg cells (1∶2000; sc-28705; Santa Cruz Biotechnology) or rabbit anti-RORγ for demonstration of Th17 cells (1∶2000; ab78007; AbCam) followed, by AlexaFluor® 555-conjugated anti-mouse (1∶1000; A31570; Molecular Probes) in the case of CD4, and by AlexaFluor® 488-conjugated anti-rabbit (1∶1000, A-21206; Molecular Probes) for Iba1. In the cases of Tbet, Foxp3 and ROR-γ after the primary antibody, sections were incubated with biotinylated anti-rabbit secondary antibody (1∶500; BA-1000; Vector Laboratories, Inc; Burlingame, CA) followed by Streptavidin-Cy3 (1∶1000; PA-43001; Amersham). Finally, sections were mounted on slides, dehydrated in graded alchohol and coverslipped in DPX. Sections were analysed using a fluorescence Nikon Eclipse E600 confocal microscope and photographed with a Leica DMIRE 2 confocal camera.

### Tissue processing for ELISA analysis

For ELISA studies, animals at different clinical scores (3 animals per score) were anesthetised using ketamine/xylazyne solution, as previously described, intracardially perfused with phosphate buffer solution (PBS) and samples corresponding to the part between the cervical and the thoracic spinal cord (C7**–**T2 levels) snap frozen in liquid nitrogen and stored at −80°C. Total protein were extracted by solubilization of spinal cords in Lysis buffer containing 250 mM HEPES (pH7.4), 1% Igepal, 5 mM MgCl_2_, 1.3 mM EDTA, 1 mM EGTA, 1 mM PMSF and protease and phosphatase inhibitor cocktails (1∶100, Sigma Aldrich). Following solubilization, samples were clarified by centrifugation at 13000 rpm for 5 min and the supernatant retained. Total protein concentration was determined with a commercial Pierce BCA Protein Assay kit (23225, Thermo Scientific) using manufacturer's protocol. Protein lysates were stored aliquoted at −80°C until their posterior used for ELISA.

### Cytokines ELISA

Cytokine ELISA kits for IFN-γ (KRC4021, Invitrogen), IL10 (KRC0101, Invitrogen), IL17 (E90063Ra, USNC Life Science Inc), IL21 (E91688Ra, USNC Life Science Inc) and Quantikine ELISA kit for IL22 (M2200, R&D Systems) were used according to the manufacturer's instruction. A standard curve was generated with each assay with the limit of detection for IFN-γ  = 13 pg/ml, IL10  = 5 pg/ml, IL17  = 6.1 pg/ml, IL21  = 5.8 pg/ml and IL22  = 3.2 pg/ml.

### CD3+ cell quantification

For CD3+ cell quantification, a total of three EAE-induced animals per clinical score and three sham were used. Analysis was performed using one entire longitudinal section of the cervical spinal cord, per animal. This section corresponds to a medial section containing both the grey and the white matter areas. Each section was photographed at 4x using a DXM 1200F Nikon digital camera joined to a Nikon Eclipse 80i microscope, and pictures taken were merged using Adobe Photoshop software. The total number of CD3+ cells per section was counted by the use of analySIS® software.

### Statistical analysis

All statistics along the study were performed using the Graph Pad Prism**®** software. Either standard two-tailed, unpaired Student's-T test, to compare two specific groups of animals, or one-way ANOVA with Tukey's post-hoc test, to compare between the different scores, were used to determine statistically significant differences.

## Results

MBP-induced Lewis rat developed the first signs of EAE around 10 days post-immunisation (dpi), displaying loss of tail tonus (score 0.5). Afterwards, during the induction phase, animals progressively presented tail paralysis (score 1) followed by hindlimb paraparesis (score 2) until reaching the maximum clinical symptomatology at score 3 (approximately around 12**–**14 dpi) when animals showed complete hindlimb paralysis. Animals remained at this maximum score for 1**–**2 days and afterwards spontaneously started to recover. During the recovery phase, animals progressively improved their mobility achieving scores 2R and 1R, and around 21 dpi, were completely recovered (score 0R) and no clinical signs were observed. During the post-recovery phase (28 dpi, 32 dpi, 40 dpi and 90 dpi), the animals did not show any visible sign of disease.

It is important to highlight here that, as we have done in previous works [3], [4], animals to be analysed for flow cytometry, ELISA and immunohistochemistry were selected not based on the days post-immunisation, but rather exclusively according to their clinical score, during the induction, peak and recovery phases. This criterion has demonstrated to be more useful because, as already stated in our previous studies, the variability among animals in the same score, if any, is very low.

### CD3+ cells

**Figure 1:**
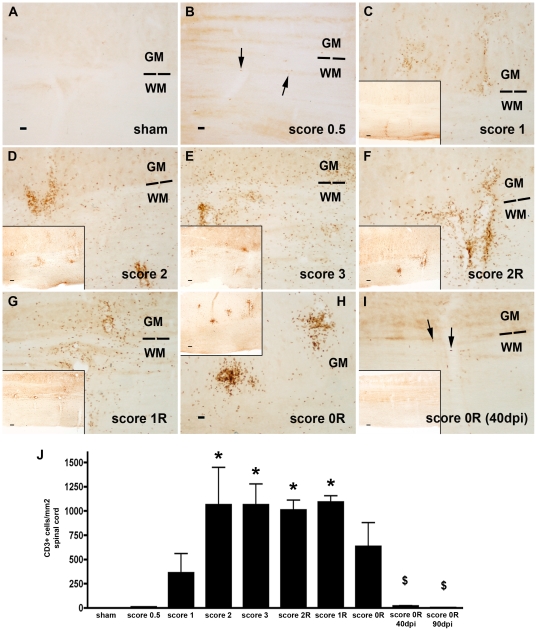
Dynamics of CD3+ cells. Immunohistochemistry for CD3 in the cervical spinal cord of sham animals (A) and in EAE animals during the induction (B**–**D), peak (E), recovery (F**–**H) and post-recovery phases (I). Arrows in B and I point to the few CD3+ cells observed in these scores. Note the progressive increase in the number of CD3+ cells in both the grey (GM) and white matter (WM) during the induction and peak phases and the maintenance of these cells during the recovery phase. Bar scale  = 30 µm. Inserts in C**–**I represents panoramic views showing the localization of CD3+ cells in the spinal cord. Bar scale = 100 µm. J) Histogramme showing analysis of CD3+ cell density observed in the different clinical scores (ANOVA and Tukey's post-hoc test *p≤0.05 with respect to sham and score 0.5; $ p≤0.05 with respect to score 2, 3, 2R and 1R).

The study of sections immunolabelled for CD3 revealed that there was no presence of T-lymphocytes in the spinal cord of sham animals (Fig. 1A). In contrast, in EAE animals, from score 0.5, few little round CD3+ cells were observed in the parenchyma (Fig. 1B). The number of CD3+ cells increased progressively during the induction phase, mainly accumulating around blood vessels (Fig. 1C, D and J), reaching their maximal at scores 2 and 3. At score 3, CD3+ cells in addition to remain in close proximity to blood vessels, were also found extensively distributed throughout the parenchyma, in both the grey and the white matters (Fig. 1E).

During the recovery phase, from score 2R to 1R, although the number of CD3+ cells remained without significant changes (Fig. 1J), the density of cells around blood vessels increased, whereas a progressive decrease in the number of CD3+ cells within the parenchyma of the cervical spinal cord was observed (Fig. 1F and G). The major part of positive cells at score 0R was accumulated in the surroundings of blood vessels of both grey and white matters (Fig. 1H). Quantitative analysis showed that at score 0R, the number of CD3+ cells apparently decreased, although the value did not reach statistical significance.

During the post-recovery phase, at score 0R**–**40 dpi, a high reduction of CD3+ cells was observed (Fig. 1J) although few perivascular CD3+ cells were still detected in the cervical spinal cord (Fig. 1I). At score 0R**–**90 dpi, CD3+ cells completely disappeared (Fig. 1J).

### CD3+CD4+cells

**Figure 2:**
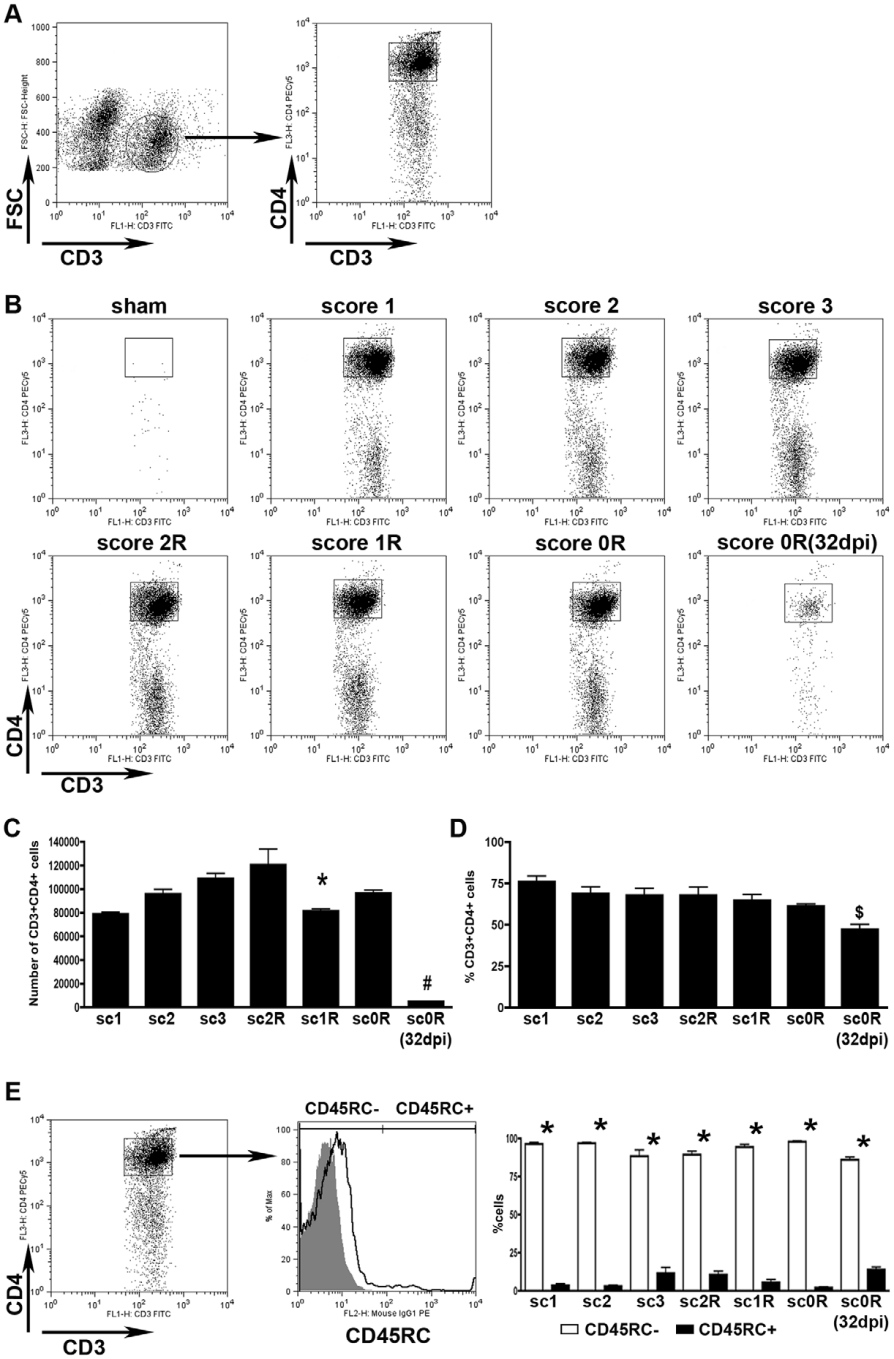
CD4 T-helper cells. A) Dot-plots exemplifying how the analysis of CD3+CD4+ cells was performed. CD3+ T-cells were gated (circle in left dot-plot) and the percentage and number of CD3+CD4+ cells were analysed in this gated population (square in right dot-plot). Spleen samples were used to determine CD3+ and CD3+CD4+ gates. B) Representative dot-plots showing the dynamics of the CD3+CD4+ cell population (square) in both sham and EAE animals during the induction phase (score 1 and 2), peak (score 3), recovery phase (score 2R, 1R and 0R) and post-recovery phase (score 0R**–**32dpi). Note that in relation to shams, there is an important and persistent population of CD3+CD4+ cells in EAE animals. C and D) Histogrammes showing the values corresponding to CD3+CD4+ cell number (C) and relative percentage (D) along EAE evolution (ANOVA and Tukey's post-hoc test, *p≤0.01 and ^#^p≤0.001 with respect to the previous score, $ p≤0.05 with respect to score 1 and score 3). E) On the left side, representative dot-plot of CD3+CD4+ cells of EAE animals. In the middle, representative histogramme where populations of CD45RC- cells (activated/effector lymphocytes) and CD45RC+ cells (naïve lymphocytes) were defined. Isotype control is represented in grey. On the right, histogramme showing the values of the percentages of CD45RC- (white columns) and CD45RC+ cells (black columns) in the different phases along EAE evolution (Student's-T test, * p≤0.0001 when compare CD45RC+ vs CD45RC- in each score). Spinal cords of each animal were analysed separately (from three to seven animals per group were used).

The number and proportion of CD4+ cells within the gated CD3+ cell population was analysed by flow cytometry along the different phases of EAE evolution (Fig. 2A). In contrast to sham animals, where no presence of CD3+CD4+ cells was found, in EAE animals, from score 1 in the induction phase, a high number of CD4+T-helper lymphocytes was observed (Fig. 2B). The number of CD3+CD4+ cells remained without significant changes along the induction and peak phases and also at score 2R, and only significantly decreased from score 1R. During the post-recovery phase, at score 0R**–**32dpi, an important and marked decrease in the number of these cells was found (Fig. 2B and C). In addition to the number of cells, we also analysed the proportion of CD3+CD4+ cells. It should be noted that CD4+ T-cells represented around 70% of the total CD3+ lymphocytes (Fig. 2D). This high percentage of CD4+ T-cells was maintained without significant changes at the different scores analysed during the induction, peak and recovery phases (Fig. S1). Only during the post-recovery phase, at score 0R**–**32dpi, a slight decrease in the proportion of CD4+ cells was observed (Fig. 2B and D).

In all phases analysed, around 90% of CD3+CD4+ cells showed a CD45RC- phenotype, characteristic of effector/memory lymphocytes, and only few CD45RC+ naïve lymphocytes were detected (Fig. 2E).

### Subtypes of CD4+ T-lymphocytes

The different subpopulations of CD4+ T-helper lymphocytes were determined by flow cytometry using specific antibodies against lineage-specific transcription factors. After gating in the CD3+ cell population, combinations of CD4 with Tbet (for Th1 cells), RORγ (for Th17 cells) and Foxp3 (for T-regulatory cells) were analysed.

#### Th1 cells

**Figure 3:**
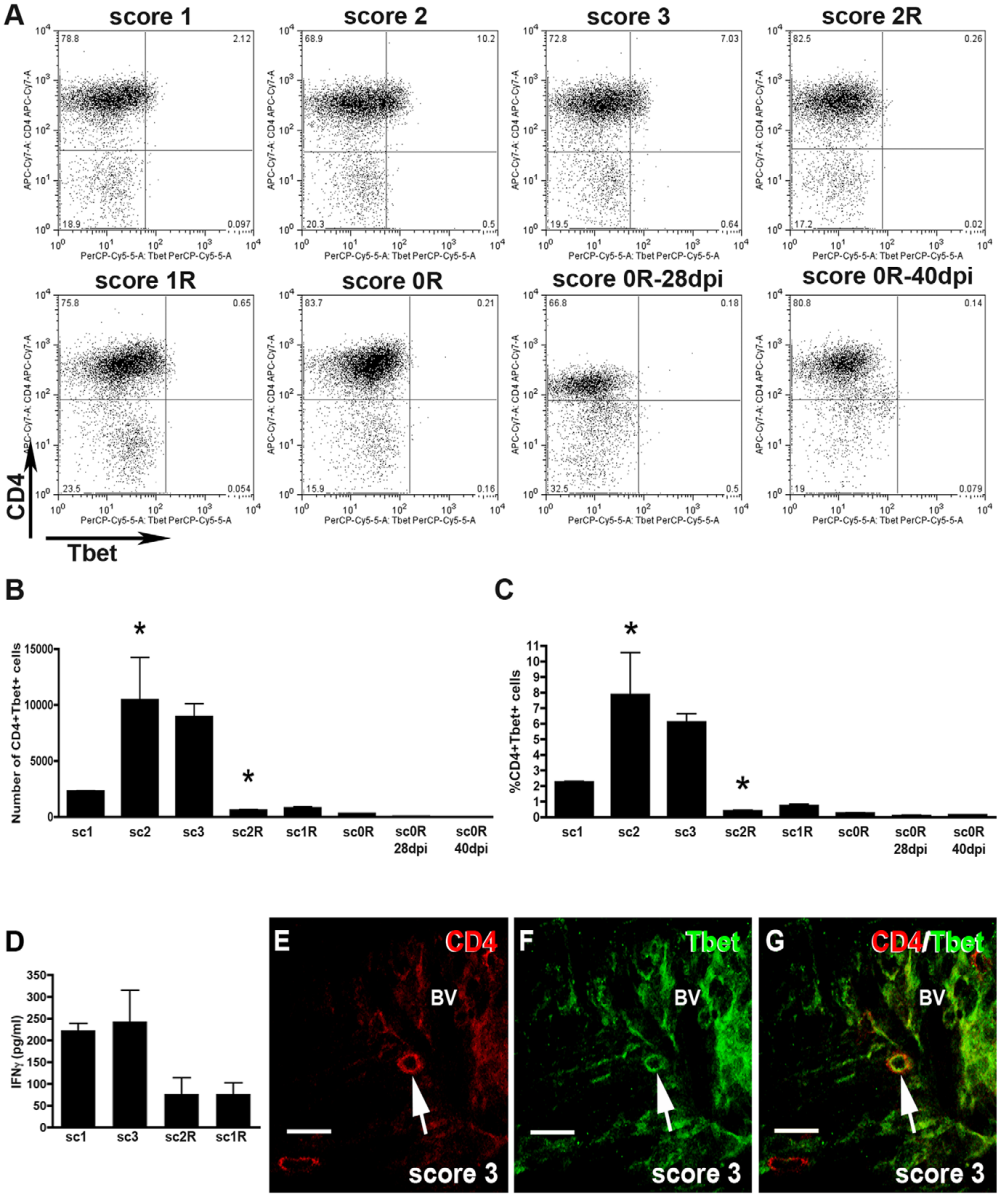
Dynamics of Th1 cell population. A) Representative dot-plots of the population of CD4+Tbet+ cells of EAE animals. Dot-plots were obtained by previously gating in the CD3+ T cell population. Different quadrants were defined by application of the appropriate isotype control. A minimum of three animals per group was pooled and three replicates per score were analyzed. B and C) Histogrammes showing, respectively, the values corresponding to the total number and the percentage of CD4+Tbet+ cell population along EAE. Note that CD4+Tbet+ lymphocytes are found during the induction and peak phases and markedly decreased at score 2R of the recovery phase (ANOVA and Tukey's post-hoc test, *p≤0.05 with respect to the previous score). D) Histogramme showing the IFNγ protein levels detected by ELISA assay at the representative scores during the induction, peak and recovery phases of EAE (ANOVA and Tukey's post-hoc test). E**–**G) Photographs of double immunostained sections showing a representative CD4+Tbet+ cell found around blood vessels (BV) (arrows point to double-immunolabelled cell). Bar scale  = 20 µm.

As shown in Fig. 3, in EAE animals, a progressive increase in the number of CD4+Tbet+ cells in the gated CD3+ cell population was detected from score 1, reaching the maximum value at scores 2 and 3 (Fig. 3A and B). From score 2R, a marked decrease in the number of CD4+Tbet+ cells was observed (Fig. 3A and B). This population of CD4+Tbet+ cells represented, at scores 2 and 3, around 7% of CD3+ lymphocytes (Fig. 3C). During the post-recovery phase (score 0R**–**28dpi and score 0R**–**40dpi) Th1 cells were absent (Fig. 3A and B). As expected, levels of IFNγ were high during the inductive phase and peak and decreased during the recovery phase (Fig. 3D).

Double immunohistochemistry demonstrated the presence of Th1 cells in the spinal cord mainly accumulated around blood vessels (Fig. 3E**–**G, Fig. S2).

#### Th17 cells

**Figure 4:**
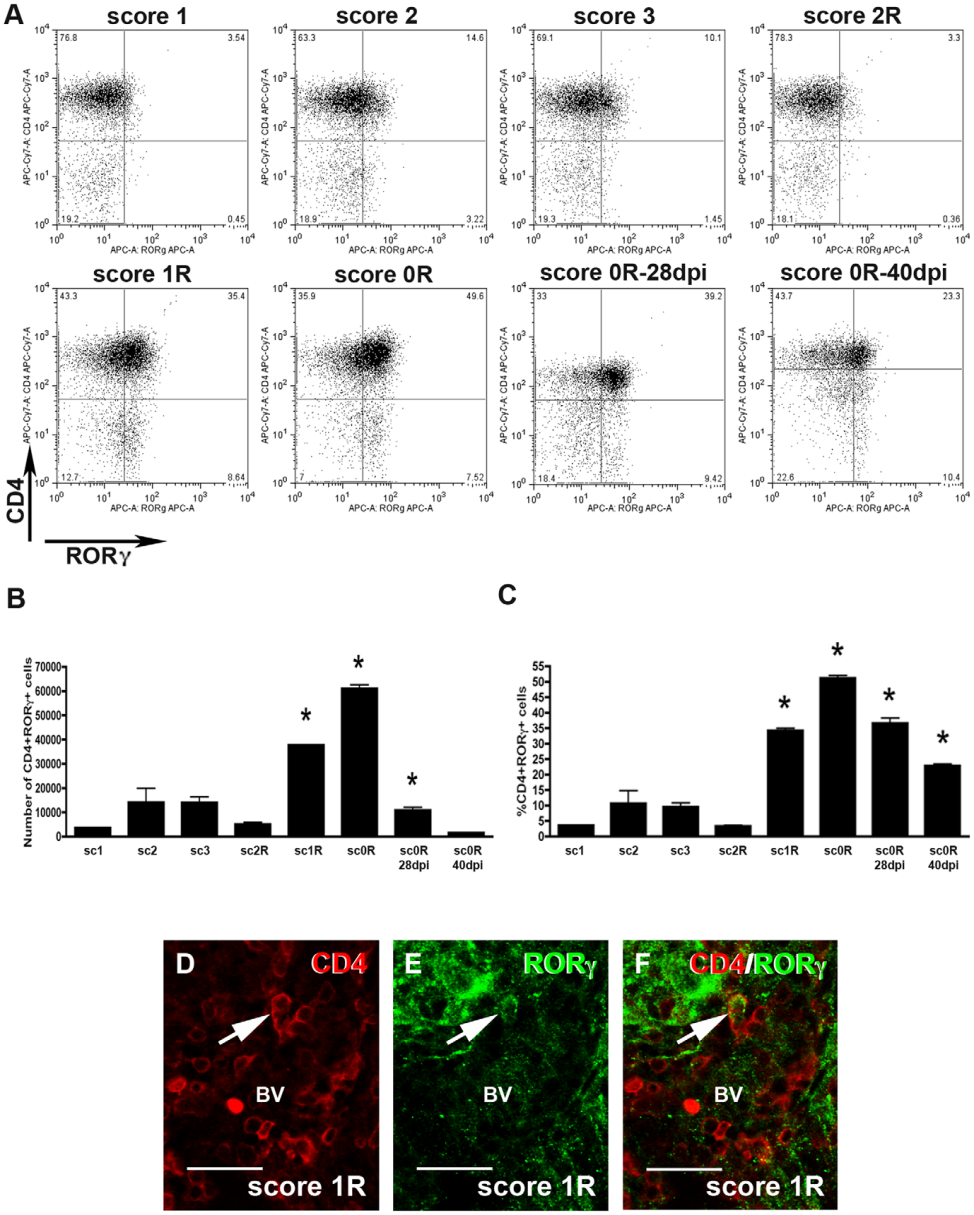
Dynamics of Th17 cells. A) Representative dot-plots of CD4+RORγ+ cells in the different phases along EAE evolution. Dot-plots were obtained after gating in the population of CD3+ T-cells. Different quadrants were defined by application of the appropriate isotype control. A minimum of three animals per group was pooled and three replicates per score were analyzed. The total number and percentage of CD4+RORγ+ cells along the different phases of EAE are represented in histogrammes B and C, respectively. Note that during the recovery and the post-recovery phases, from score 1R, a great increase in the subpopulation of CD4+RORγ+ cells was observed (ANOVA and Tukey's post-hoc test, *p≤0.001 with respect to the previous score). D**–**F) Photographs of double immunolabelled sections showing a representative CD4+RORγ+ cell (arrow) observed around blood vessels (BV). Bar scale  = 30 µm.

In EAE animals, some CD4+RORγ+ cells were observed during the induction phase, the peak and at score 2R in the recovery phase (Fig. 4A and B). The number of CD4+RORγ+ cells highly increased from score 1R in the recovery phase, reaching the maximum value at score 0R (Fig. 4B). During the post-recovery phase, an important decrease in the number of CD4+RORγ+ cells was detected (Fig. 4B). In terms of percentage of this population, it should be noted that, whereas during the induction and peak phases the subpopulation of CD4+RORγ+ lymphocytes represented around 7% of CD3+ T-cells, from score 1R in the recovery phase the percentage of these lymphocytes increased until a value of around 50% at score 0R (Fig. 4C). During the post-recovery phase, a decrease in the percentage of these cells was found although levels remained higher than those observed during the induction and peak phases (Fig. 4C). In addition to CD4+RORγ+ cells, our analysis also demonstrated the presence of a population of CD4-RORγ+ cells (less than 10%) (Fig. 4A).

The study of double immunolabelled sections indicated the presence of CD4+RORγ+ cells mainly accumulated around blood vessels in the spinal cord of EAE animals (Fig. 4D**–**F, Fig. S2).

#### T-regulatory cells

**Figure 5:**
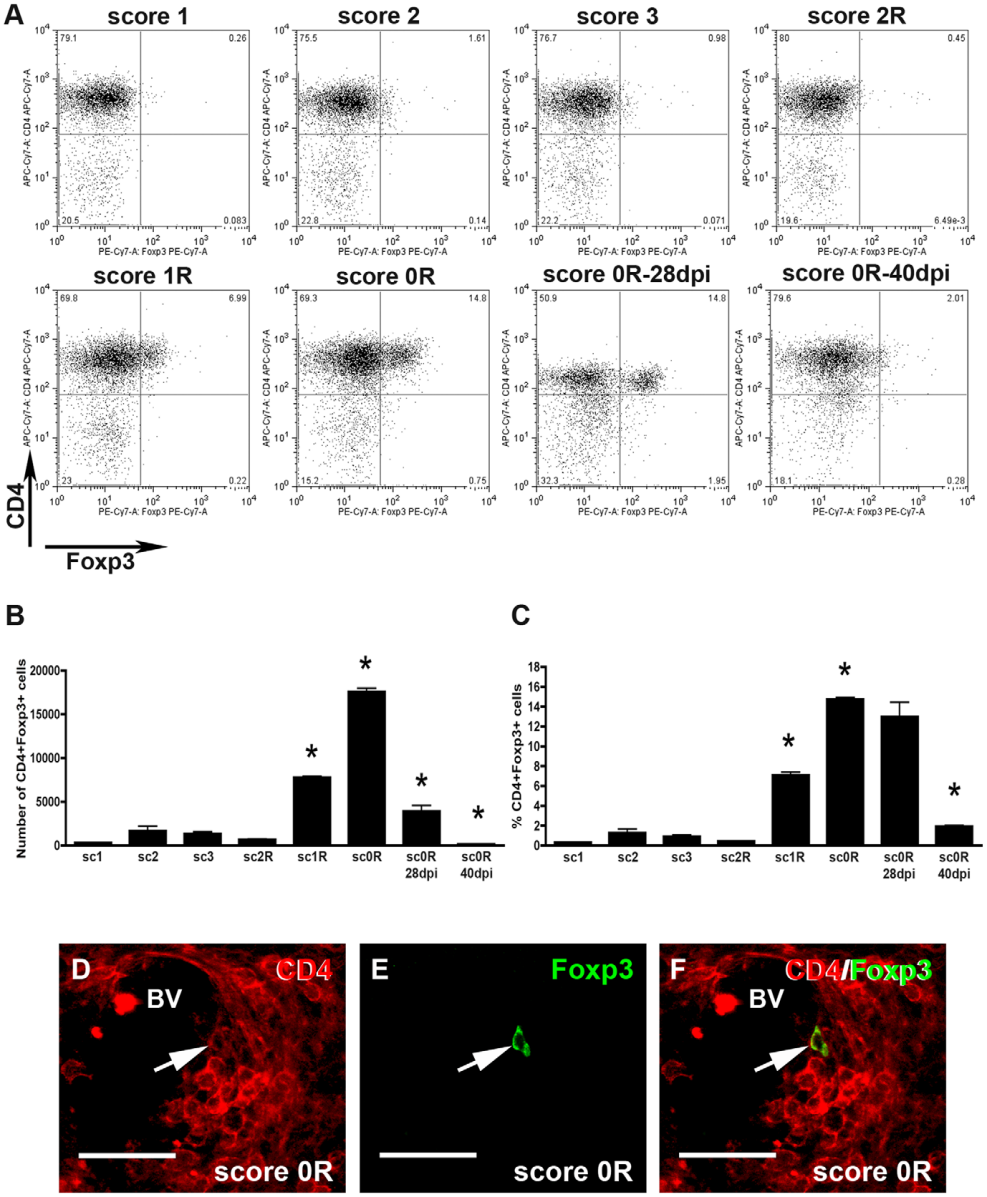
Dynamics of T-regulatory cells. A) Representative dot-plots of the CD4+Foxp3+ cell population along the different phases of EAE evolution. Dot-plots were obtained after gating in the population of CD3+ T-cells. Quadrants were defined by application of the appropriate isotype control. A minimum of three animals per group was pooled and three replicates per score were analyzed. B and C) Histogrammes showing, respectively, the total number and percentage value of CD4+Foxp3+ cells along the different phases of EAE. Note that although the number of CD4+Foxp3+ cells decreased at 0R**–**28dpi, their percentage remained high until score 0R**–**40dpi (ANOVA and Tukey's post-hoc test, *p≤0.001 with respect to the previous score). D**–**F) Photographs of double immunolabelled sections showing a representative CD4+Foxp3+ cell (arrows) found around blood vessels (BV). Bar scale  = 30 µm.

As shown in Fig. 5, during the induction phase, the peak, and at score 2R in the recovery phase, only few CD4+Foxp3+ cells within the gated CD3+ population were observed (Fig. 5A and B). From score 1R during the recovery phase, a substantial increase in the number of CD4+Foxp3+ cells was observed until score 0R, when the maximum quantity of these cells was found (Fig. 5A and B). The number of these CD4+Foxp3+ cells significantly decreased during the post-recovery phase from score 0R**–**28dpi to score 0R**–**40dpi (Fig. 5B). This population of CD4+Foxp3+ cells represented less than 10% of CD3+ T-cells at score 1R, and approximately 15% at score 0R (Fig. 5C). Remarkably, at score 0R**–**28dpi, the proportion of these cells remained unchanged, though the total number of CD4+Foxp3+ cells decreased considerably (Fig. 5C). It was not until score 0R**–**40dpi when the proportion of this population underwent a substantial decline (Fig. 5C).

Double immunofluorescence analysis showed the accumulation of T-reg cells around blood vessels (Fig. 5D**–**F).

### Expression of Th17 and T-reg related cytokines

**Figure 6:**
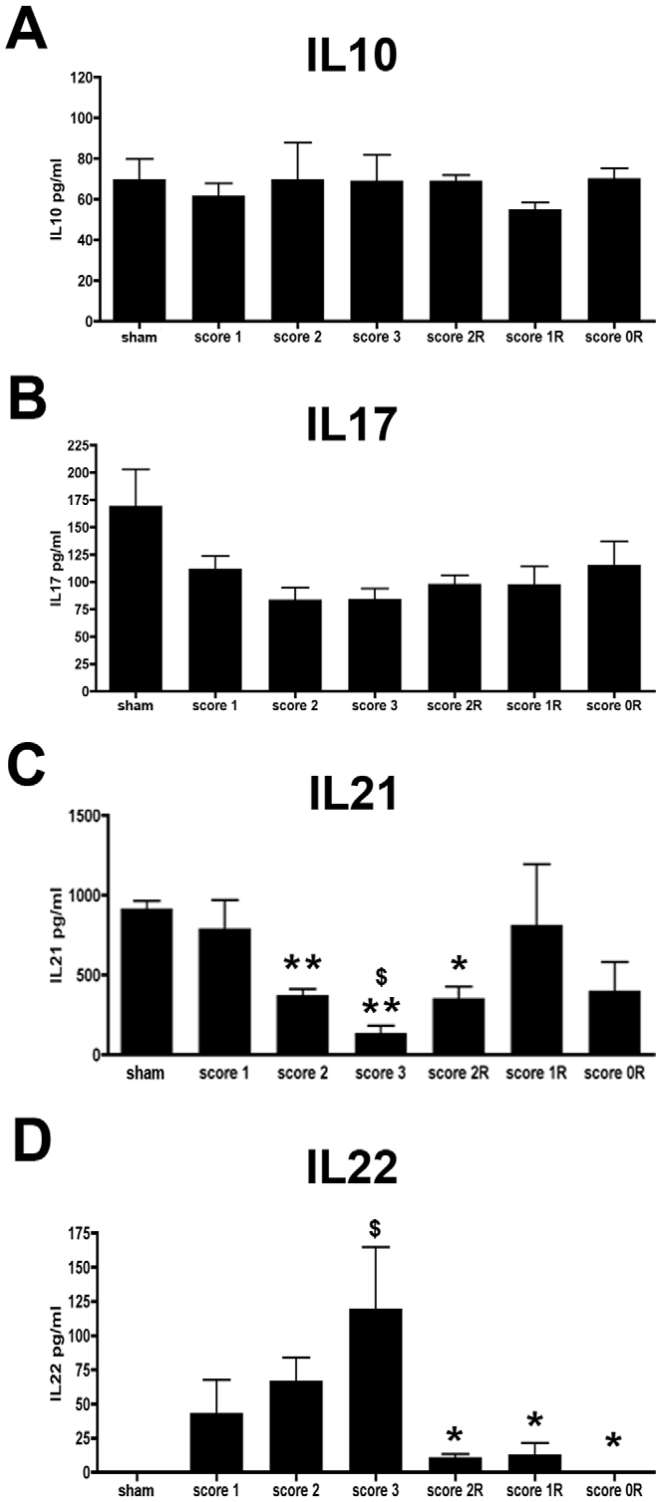
Cytokine profile along EAE. Histogrammes showing the IL10 (A), IL17 (B), IL21 (C) and IL22 (D) protein levels along the different phases of EAE. Note that while IL10 and IL17 cytokines remained unaltered along EAE evolution (A and B), IL21 levels decreased in score 2, 3 and 2R (C) (Student's-T test, **p≤0.01 and *p≤0.05 with respect to sham; $ p≤0.05 with respect to score 1 and 2) and IL22 levels were higher during the inductive phase and markedly decreased during the recovery phase (D) (ANOVA and Tukey's post-hoc test, $ p≤0.05 with respect to sham; *p≤0.05 with respect to score 3).

The analysis of different cytokines such as IL17, IL21 and IL22 commonly related with the subpopulation of Th17 lymphocytes, as well as IL10, related with both Th17 and T-reg populations were studies using ELISA assays. Our results indicated that interestingly, there are no changes in IL10 and IL17 protein levels along the different phases of the disease (Fig. 6A and B). In contrast, a decrease in IL21 was found from score 2 of the inductive phase of EAE showing the lowest levels of expression of this phase at score 3 (Fig. 6C). A slight increase of this cytokine was found during the recovery phase although it was only at score 1R when the level of expression was similar to the observed in sham animals.

The analysis of IL22 indicated that levels of this cytokine increased in the induction phase showing a high level of expression at the peak of disability (score 3). Subsequently, a marked decrease of IL22 was found since score 2R in the recovery phase. Like in sham animals, levels of this protein were undetectable at score 0R (Fig. 6D).

## Discussion

In this study a detailed analysis of the different subtypes of T-helper lymphocytes that infiltrated the CNS along the diverse phases of the acute EAE model in Lewis rat is performed. Our results show that during the induction and peak phases, the number of CD3+ cells increased in close relationship to disease severity. Nevertheless, during the recovery phase, although clinical signs decreased, the number of CD3+ lymphocytes remained very high without significant variations. Even during the post-recovery phase, some CD3+ cells were still found within the parenchyma of the spinal cord. Similarly we found that the proportions of CD4+ T-helper cells remained unchanged along the evolution of EAE, displaying a phenotype of activated/effector cells (CD45RC- cells) throughout all phases analysed. These findings are striking as traditionally T-lymphocytes, especially those with a CD4+ T-helper phenotype, are considered pathogenic cells in EAE. This assumption is partly due to the capacity of CD4+ lymphocytes to induce the disease when injected into susceptible animals [24] and their presence at the peak of the disease in different models of EAE [18], [21], [22], and partly due to studies reporting an improvement of clinical symptomatology of EAE after treatments that produced a decrease in lymphocyte infiltration [25]. Although in this line, a decrease in the number of these cells could be also expected during the spontaneous recovery phase of this acute EAE model, noticeably, the findings obtained in this study show that the number of CD4+ T-lymphocytes remained elevated during this phase. These observations correlate with our previous findings showing that microglia/macrophages in this EAE model remained activated also during the recovery and post-recovery phases of this acute EAE model [3], [4], and point towards a cross-talk between microglia and lymphocyte populations along the evolution of the disease, not only during the induction and peak, but also during the recovery phase [26].

It is important to take into account that, CD4+ T-cells comprise a wide range of subpopulations which not only play pathogenic functions but also may play regulatory/suppressive roles [7], [8], [9], [27] that may explain why in our study, these CD4+T cells remained high also during all the recovery phase. In this sense, by using lineage-specific transcription factors, we carried out an accurate study of different subsets of CD4+ T-helper lymphocytes along EAE evolution, determining the specific temporal pattern of infiltration of Th1 (Tbet+), Th17 (RORγ+) and T-reg (Foxp3+) cells. These specific transcription factors were shown to regulate genes encoding the signature cytokines of these different subpopulations of T-cells. Thus, Th1 cytokines such as IFN-γ and TNF-α are regulated by Tbet [28], Th17 cytokines IL17, IL22 or IL21 by RORγ and cytokines associated with T-reg cells such as IL10 and TGF-β were regulated by Foxp3 [29]. Our findings revealed that these different subsets of T-helper lymphocytes are present in the spinal cord in specific phases along the course of the disease. In our study it was clearly demonstrated that the number of Th1 lymphocytes (CD3+CD4+Tbet+ cells) and the expression of the pro-inflammatory cytokine IFNγ, parallels the disease evolution, increasing progressively during the induction phase, reaching the maximum at the peak of the disease and decreasing thereafter during the recovery phase.

In parallel to a decrease in Th1 cells, during the recovery phase, a high increase in Th17 and T-reg cell populations was found. Th17 cells are commonly considered as a pathogenic population of lymphocytes, as they have been detected at the onset of EAE in mice [30], infiltrated the CNS parenchyma after the initial influx of Th1 lymphocytes [27], [31] and, when injected into susceptible animals, they are able to induce EAE [12], [31]. However in our study, the number of these cells greatly increased at the end of the recovery phase (from score 1R), peaking at score 0R. One plausible explanation for these distinct results could be the differences in the EAE model used. In contrast to the aforementioned studies describing Th17 cells as pathogenic lymphocytes, which used relapsing-remitting or chronic models induced in mice, in this study we analysed the acute EAE model induced in Lewis rats. This model has a unique feature: the complete and spontaneous recovery, that was absent in both the relapsing-remitting or chronic models in mice, and whose nature has not yet been established. Thus, it is reasonable to think that cells involved in the evolution of this acute model, including Th17 cells, can play different roles in the progressive or chronic models of EAE. Indeed, this is the first study describing the specific dynamics of different subsets of T cells in correlation to clinical symptomatology in an acute EAE model with spontaneous recovery. Therefore, the pathogenic role attributed to Th17 cells in mice, could be not applicable to this acute rat model. In fact, the exact role played by Th17 cells in autoimmunity has been under active discussion [32], specially after the publication of some interesting studies providing data that bring in question the pathogenic potential of IL17, the signature cytokine of Th17 cells. In one hand IL17 treatment has been shown to induce an amelioration of experimental autoimmune uveitis in Lewis rats [33] and, in the other hand, mice with a conditional deletion of IL17 develop EAE normally [34]. In agreement with this last statement, the analysis of IL17 protein levels in our study demonstrated that despite the high increase in Th17 lymphocytes during the recovery phase, protein levels of this cytokine remained unaltered along the different phases of EAE, suggesting that may be IL17 is not the key cytokine secreted by these lymphocytes in this acute model or that their function is not as relevant as though. In this way, it has been demonstrated that beyond to produce pro-inflammatory cytokines such as IL17, Th17 lymphocytes are also able to secrete anti-inflammatory cytokines such as IL10 [35] with a suggested beneficial effect specifically in acute EAE in Lewis rat [36], and others such as IL22 [9] and IL21 whose role in EAE is still not well established. Addition of IL21 before the onset of EAE symptoms aggravates the disease [37], but the blockade of IL21/IL21R pathway induced an enhancement of EAE severity [38], [39]. Furthermore, exposure of dendritic cells to IL21 induced an immature phenotype of these cells [40] that cannot induce T-cell responses [41], [42]. In this regard, it is interesting to highlight that we have previously reported that parenchymal microglial cells acquire an immature DC phenotype during the recovery phase, characterised by the expression of CD1 (an immature marker of dendritic cells) and MHCs but not co-stimulatory molecules [3]. Thus, we can speculate that cytokines secreted by Th17 lymphocytes during the recovery phase can be involved in the induction of changes in microglial phenotype during this phase. Following this hypothesis, we analysed the pattern of expression of IL10, IL21 and IL22 along the different phases of acute EAE. Interestingly, in contrast to our initial hypothesis and as already mentioned for IL17, the pattern of expression of these three cytokines did not correlate with the presence of Th17, indicating together that, at list in this acute EAE model, Th17 cells are not producing IL17, IL10, IL21 or IL22. It is interesting to remark the marked decrease observed in the levels of IL22 in the initiation of the recovery phase. This striking result open a new and interesting way of study pointing to this cytokine as a putative key factor involved in the evolution of EAE. One possibility is that this cytokine may drive the inflammatory events occurring during the inductive and peak phases, and therefore a decrease in IL22 production may lead to stop inflammation and initiate the recovery. In another hand, also we can argue that certain levels of this cytokine are required to initiate the recovery phase, therefore when reach the correct threshold triggers the recovery mechanisms. Further studies in this sense are however necessary to completely understand the role played by this cytokine in the acute EAE model in Lewis rat. At this time, it is also interesting to point out that in general, there is a lack of information in the literature regarding the specific pattern of cytokine expression along the different phases of the different EAE models. Moreover, most studies linking lymphocytes with the secretion of various cytokines are based on the isolation of these cells and their subsequent activation in vitro, results that indicate the ability of these lymphocytes to produce these cytokines but, as already demonstrated in this study, does not necessarily implicate that they are doing the same function in vivo in the CNS. A better understanding of the real scenario occurring within the CNS parenchyma in terms of cytokine profile may be very helpful to understand the processes leading to the resolution and/or chronicity of this disease in the different animal models.

In addition to the Th17 lymphocyte population, during the recovery phase we also found a significant increase in the number of Foxp3+ T-reg cells. Accumulation of these T-regs in the CNS has already been reported during recovery in mice EAE models [14], [15], [43], albeit to our knowledge this is the first study demonstrating accumulation of Foxp3+ cells within the spinal cord of acute EAE-induced rats. Some studies have demonstrated the beneficial role played by these cells in EAE pathogenesis in mice. As such, injection of Foxp3+ T-reg cells, derived from EAE-recovered mice or in vitro-expanded, ameliorates EAE symptomatology when injected into MOG-induced mice [15], [44]. In the same way, a decrease or inactivation of Foxp3+ cell numbers in vivo by the use of anti-CD25 antibody treatment, makes these treated animals more vulnerable to EAE induction [45]. Since we found the major proportion of T-reg cells during the recovery phase of EAE, we can speculate that Foxp3+ cells in this model may play also a role in the resolution of the immune response and can be one of the critical factors involved in the spontaneous recovery characteristic of the model.

Noticeably, during the post-recovery phase, although the number of both Th17 and T-reg cells declined, the proportion of these cell populations remained high, mostly at score 0R**–**28dpi. This long-time permanence suggests that these cells can still play an active role even after the animals have fully recovered and do not show any clinical symptom, may be being involved in the tolerance mechanism that, after EAE induction, renders these Lewis rats resistant to further immunization with the same antigen [1].

Several studies have demonstrated that recovery from acute EAE is commonly associated with apoptotic elimination of pathogenic lymphocytes [46], [47], [48], [49]. As our findings showed that when the recovery phase started the number of Th1 cells abruptly decrease, we hypothesise that this decrease may be due to an induced apoptotic elimination of these lymphocytes. In fact, we detected a high amount of apoptotic lymphocytes, especially during the peak of the disease, in near proximity to microglial cells (Fig. S3). This hypothesis fits well with our previous findings [3] showing that during the induction and peak phases, microglial cells displayed an immature dendritic-cell phenotype (MHC-class I and II+/CD1+/B7.1-/B7.2-) which may provide an anergic or apoptotic signal to the Th1-infiltrated lymphocytes [26] and is in agreement with a recently published study [50] showing that myeloid-derived suppressor cells, an heterogeneous population of immature myeloid cells involved in the regulation of immune responses in tumour microenvironments [51], can induce the apoptosis of infiltrated T-cells also in a chronic mouse model of EAE. Nevertheless, we cannot exclude the possibility that the different T-cell populations observed and their dynamics are the result of a phenomenon of lymphocytic plasticity, bearing the interconversion between T-cell subtypes, as has recently been postulated by some authors [52], [53].

### Conclusion

In conclusion, we clearly demonstrate in this study that, although the number of T-lymphocytes inside the spinal cord parenchyma remains constant along the three main phases of EAE (induction, peak and recovery) the lymphocytic phenotype along the different phases undergo major changes. During the induction and peak phases major part of lymphocytes exhibited a phenotype of Th1 cells, whereas during the recovery and post-recovery phases T-lymphocytes mainly displayed a phenotype of Th17 or T-reg cells. Moreover, our results demonstrated a specific cytokine profile along the different phases of this acute EAE model, which differs from the described in chronic and relapsing-remitting models, characterized by no changes of IL10 and IL17 levels, decrease of IL21 on the peak phase, and high levels of IL22 during the induction and peak phases that markedly decrease during recovery.

These results suggest that the general view of lymphocyte infiltration in the CNS as a detrimental process should be reviewed. Our results demonstrated that it is not a question of presence or absence of T-cells, but the scenario is more complicated, being necessary to consider the specific subtype of infiltrated lymphocytes, their function and the specific interactions that these lymphocytes established with resident cells within the CNS. Further studies to analyse these interactions are necessary to understand the specific role played by these lymphocytes along EAE evolution. In this context, actions played by secreted cytokines in particular situations should also be more carefully reviewed.

## Supporting Information

Figure S1 **A–D) Cells immunolabelled with CD3 and CD4 were observed at the different scores of EAE evolution (arrows).** Note that, in addition to these double positive cells, also few CD3+CD4- cells (arrowheads in B and D) and CD3-CD4+ cells (asteriscs in A, B and C) were also observed. Bar scale  = 30 µm
(TIF)

Figure S2 **A–C) Photographs showing the CD4 (A) and Tbet (B) immunostatining at score 0R.** Note that the few Tbet+ cells did not colocalize with CD4 (arrows in A**–**C). D**–**F) Colocalization between CD4 and ROR-γ was found at score 0R in some cells located near blood vessels (arrows). Bar scale  = 30 µm
(TIF)

Figure S3 **Apoptotic lymphocytes**. A**–**C) Double immunolabelling combining the microglial marker Iba1 and CD4, and counterstained with DAPI. Iba1+ microglial cells (green) were observed closely related to apoptotic cells that were identified as CD4+ lymphocytes (arrows in A, B and C). Bar scale  = 20 µm
(TIF)
