# Supplementary figures and images for: Increase in Th17 and T-reg Lymphocytes and Decrease of IL22 Correlate with the Recovery Phase of Acute EAE IN Rat
Source: PLoS One. 2011 Nov 7;6(11):e27473. doi: 10.1371/journal.pone.0027473 (PMC3217052; doi:10.1371/journal.pone.0027473)

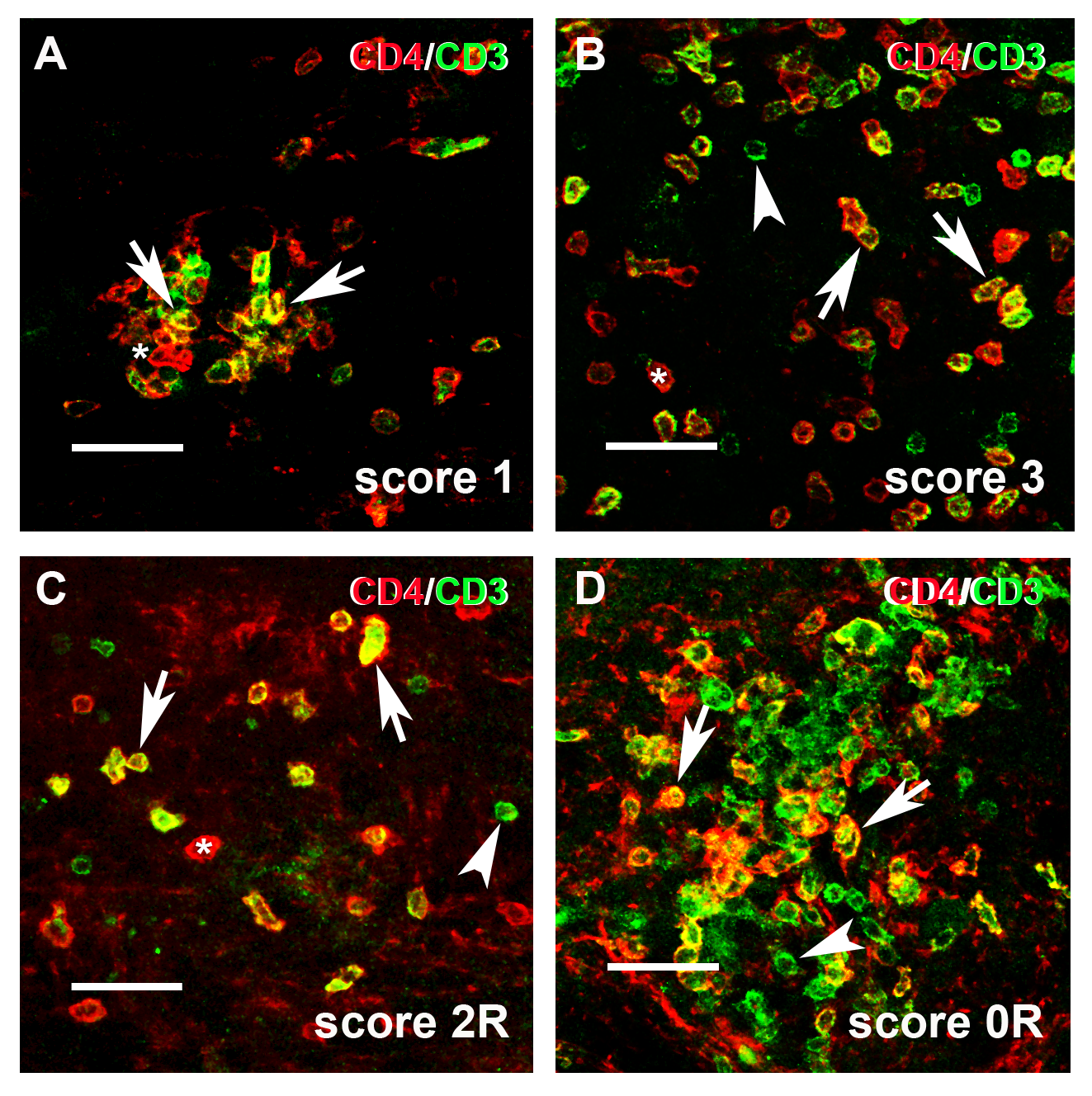

Supplement: Figure S1 — A–D) Cells immunolabelled with CD3 and CD4 were observed at the different scores of EAE evolution (arrows). Note that, in addition to these double positive cells, also few CD3+CD4- cells (arrowheads in B and D) and CD3-CD4+ cells (asteriscs in A, B and C) were also observed. Bar scale = 30 µm (TIF) [file pone.0027473.s001.tif]

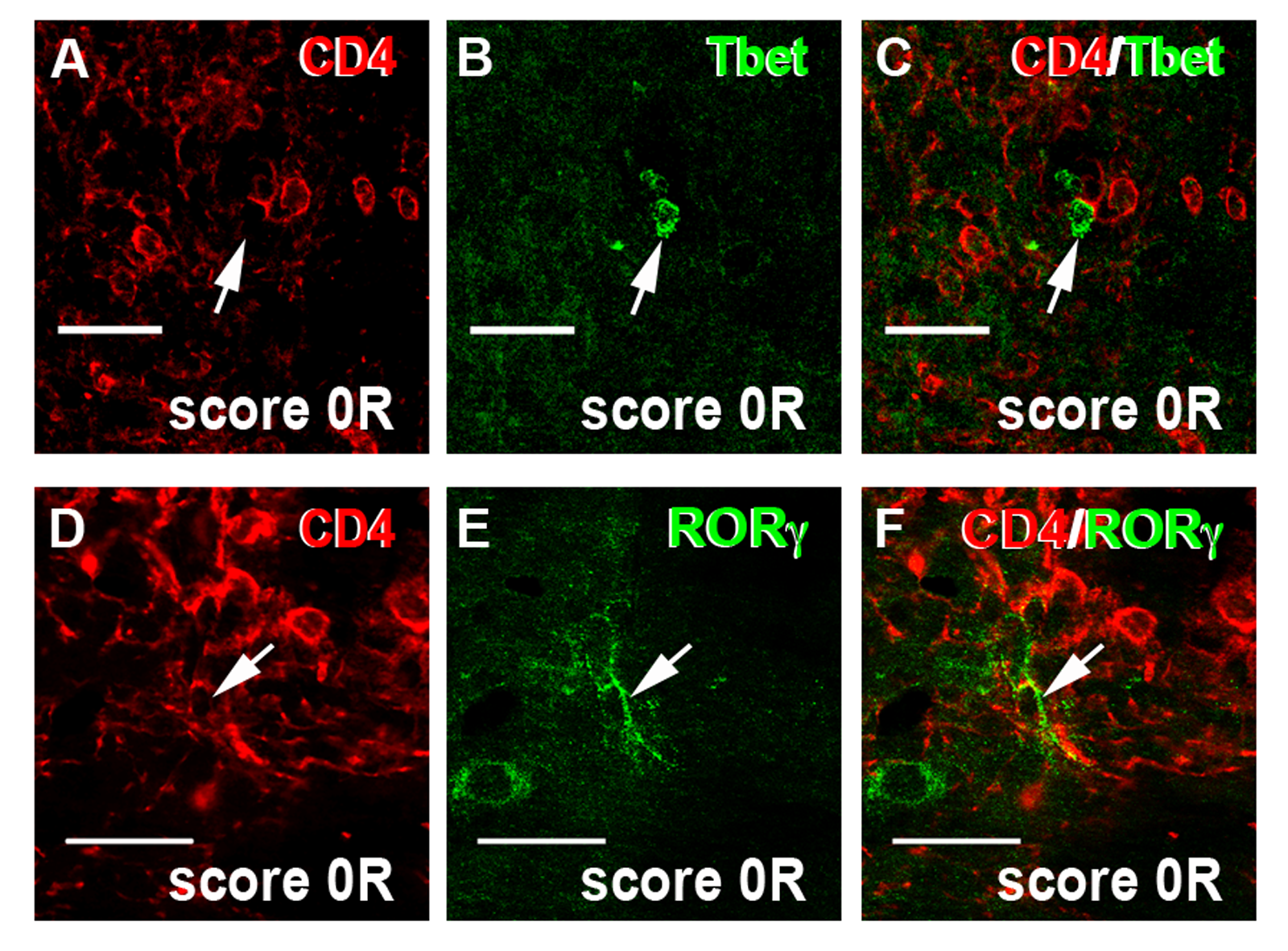

Supplement: Figure S2 — A–C) Photographs showing the CD4 (A) and Tbet (B) immunostatining at score 0R. Note that the few Tbet+ cells did not colocalize with CD4 (arrows in A–C). D–F) Colocalization between CD4 and ROR-γ was found at score 0R in some cells located near blood vessels (arrows). Bar scale = 30 µm (TIF) [file pone.0027473.s002.tif]

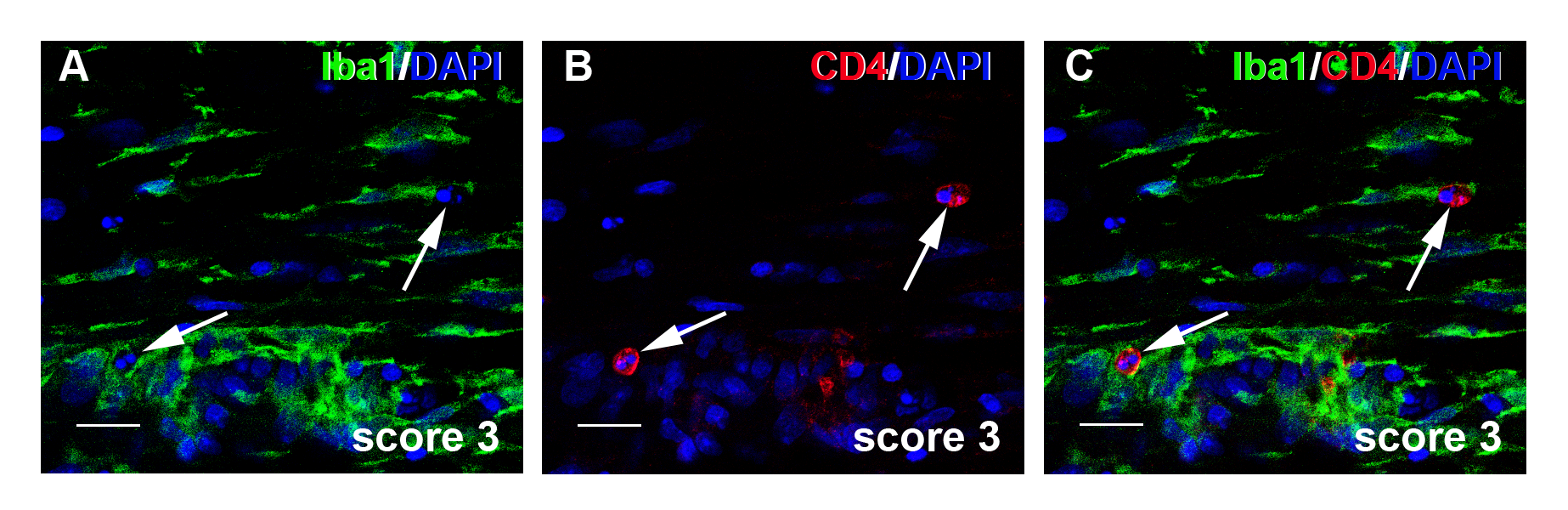

Supplement: Figure S3 — Apoptotic lymphocytes. A–C) Double immunolabelling combining the microglial marker Iba1 and CD4, and counterstained with DAPI. Iba1+ microglial cells (green) were observed closely related to apoptotic cells that were identified as CD4+ lymphocytes (arrows in A, B and C). Bar scale = 20 µm (TIF) [file pone.0027473.s003.tif]
